# A divergent approach for the synthesis of (hydroxymethyl)furfural (HMF) from spent aromatic biomass-derived (chloromethyl)furfural (CMF) as a renewable feedstock†

**DOI:** 10.1039/d0ra09310f

**Published:** 2020-12-21

**Authors:** Mangat Singh, Nishant Pandey, Bhuwan B. Mishra

**Affiliations:** Center of Innovative and Applied Bioprocessing (CIAB) Sector 81 (Knowledge City), S.A.S. Nagar Mohali-140306 Punjab India bhuwan@ciab.res.in +91-172-5221541; Department of Chemistry, Faculty of Science, Panjab University Chandigarh-160014 India

## Abstract

Extraction of commercial essential oil from several aromatic species belonging to the genus *Cymbopogon* results in the accumulation of huge spent aromatic waste which does not have high value application; instead, the majority is burned or disposed of to vacate fields. Open burning of spent aromatic biomass causes deterioration of the surrounding air quality. Therefore, a new protocol has been developed for chemical processing of spent biomass to obtain 5-(chloromethyl)furfural (CMF) with high selectivity (∼80%) and yields (∼26 wt% or ∼76 mol% with respect to pre-treated biomass) *via* refluxing in aqueous HCl in the presence of NaCl as a cheap catalyst. No black tar formation and gasification were observed in the processing of the spent aromatic biomass. Spent aromatic waste-derived CMF was further converted to 5-(hydroxymethyl)furfural (HMF) in good yields by a novel one pot method using iodosylbenzene (PhIO) as a reagent under mild reaction conditions.

## Abstract 2

Extraction of commercial essential oil from aromatic crops results in the accumulation of huge spent aromatic waste which can be used for production of platform chemicals such as xylose, CMF and HMF.

## Graphical abstract

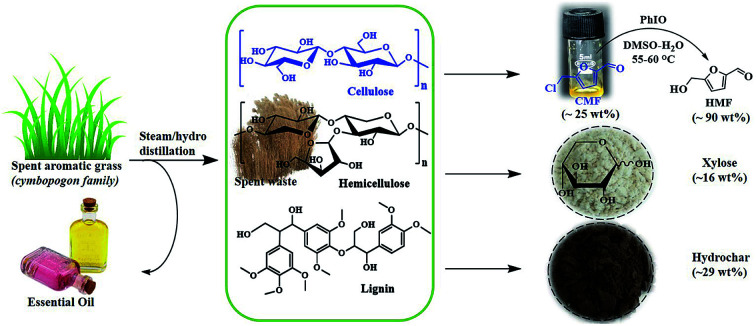

## Introduction

There are about 400 plant species being cultivated worldwide on a large commercial scale for the production of essential oils. Among these, lemongrass (*Cymbopogon flexuosus*), citronella grass (*C. winterianus*) and palmarosa (*C. martini*) are economically most popular in the tropical and subtropical regions of Asia, Africa and America. Extraction of essential oil from these aromatic crops results in the accumulation of huge spent aromatic waste as a by-product. Global industrial processing of lemongrass alone accounts for approximately 30 000 000 tons per annum of aromatic waste.^1^ India, being a worldwide leader of citronella and mentha essential oil, generates approximately ∼6.0 million tons per annum of spent aromatic waste. This most abundant and underutilized biomass does not have high value application, hence the majority is burned or disposed of to vacate fields. Open burning of spent aromatic biomass causes deterioration of the surrounding air quality due to release a substantial amount of pollutants into the atmosphere such as trace gases (*e.g.*, carbon dioxide, carbon monoxide, methane *etc.*), volatile organic compounds (*e.g.*, monoterpenes, sesquiterpenes, phenols, oxides, esters, aldehydes *etc.*), precursors of O_3_ and aerosols, especially organic carbon (OC) and black carbon (BC).^2^

Spent aromatic biomass contains varying composition of hemicellulose, cellulose, and lignin depending on the source, species, and region of the cultivation. Besides having a high calorific value, the high cellulose (35–40%) and hemicellulose (25–30%) content makes it a suitable biomass for bioenergy production. Terpenoids obtained from the aromatic crops have been found economic and fungible with the existing liquid fuels due to low temperature operable properties.^3^ Ethanol production from spent aromatic biomass has also been prospected, however, residual volatiles *e.g.* citral, geraniol, limonoids, *etc.*, in the spent waste may inhibit the growth of *Saccharomyces cerevisiae* during microbial fermentation in biorefineries.^4^ The biomass has been also investigated for supply of nutrients to the other crops, nutrient recycling for fertilizer economy,^5^ and for the production of high value chemicals, *e.g.*, xylose, levulinic acid, lignin, *etc.*, by the chemical processing.^6^

Synthesis of 5-(hydroxymethyl)furfural (HMF) and its derivatives have recently been advanced as a highly promising platform compounds for easy access to a range of renewable chemicals and materials.^7^ However, the HMF has only been produced from fructose at pilot scales, and to date no scalable approach for its production from raw biomass has been reported.^8^ Frequent water solubility, high boiling point, and sensitivity to the acidic conditions impose difficulties in large scale production of HMF from carbohydrates. Therefore, a potentially disruptive innovation in the arena of renewable chemicals has recently been introduced in the form of the HMF analog, 5-(chloromethyl)furfural (CMF) which unlike HMF, can be obtained in high yield and purity from glucose or even directly from the cellulosic biomass (Table 1). CMF being hydrophobic in nature, can readily be isolated from reaction media, and converted further into different compounds, *e.g.*, 2,5-dimethylfuran (DMF), 5-ethoxymethylfurfural (EMF), HMF, *etc.*, due to the interesting reactive chemistry.^9–12^ Conventionally, CMF is prepared by the treatment of HMF or cellulose with dry hydrogen halide, wherein, the hydroxyl group in HMF undergoes substitution by a halogen atom.^13–15^ Other methods involve, treatment of carbohydrate with HCl–LiCl,^11,16^ deep eutectic solvents (such as choline chloride),^17,18^ HCl–H_3_PO_4_ in presence of CHCl_3_ under biphasic condition,^19^ and metal chlorides (such as CrCl_3_, AlCl_3_ and ZnCl_2_) or mixed metal chlorides (CrCl_3_–ZnCl_2_).^20^ Notwithstanding the numerous efforts depicted above, each of them suffers from at least one of the following limitations: low product selectivity, formation of by-products, low conversion efficacy and product yield, harsh reaction conditions, requirement of costly reagents, prolonged reaction times and tedious operations with complex setups (facility for continuous extraction). These drawbacks seriously hamper their potential applications in industries and warrant searching for more general and efficacious route for CMF synthesis from lignocelluloses.

**Table 1:** Carbohydrate conversion to CMF reported in literature^a^

Entry	Substrate	Catalyst	Temperature (°C)	Time (h)	Solvents	CMF yield (mol%)	Ref.
1	Corn stover	HCl	100	3	DCE	70.0	10
2	Cellulose	HCl + LiCl	65	>18	DCE	71.0	11
3	Chitin	HCl	150	1	DCE	44.5	12
4	Fructose	ChCl + AlCl_3_	120	5	MIBK	50.3	18
5	Inulin	ChCl + AlCl_3_	120	5	MIBK	22.6	18
6	Sucrose	ChCl + AlCl_3_	120	5	MIBK	17.8	18
7	Eucalyptus kraft pulp	HCl + H_3_PO_4_	45	20	CHCl_3_	21.3	19
8	Norway spruce soft wood TMP	HCl + H_3_PO_4_	45	20	CHCl_3_	33.7	19
9	Eucalyptus hard wood	HCl + H_3_PO_4_	45	20	CHCl_3_	47.4	19
10	Bamboo pulp	HCl + ZnCl_2_ + CrCl_3_	40	10	CHCl_3_	32.7	20
11	Eucalyptus pulp	HCl + ZnCl_2_ + CrCl_3_	40	10	CHCl_3_	36.2	20
12	Bagasse pulp	HCl + ZnCl_2_ + CrCl_3_	40	5	CHCl_3_	50.1	20
13	Palmarosa	HCl + NaCl	100	1	CHCl_3_	76.5	This work
14	Lemon grass	HCl + NaCl	100	1	CHCl_3_	72.4	This work
15	Citronella grass	HCl + NaCl	100	1	CHCl_3_	65.8	This work

a DCE, dichloroethane; MIBK, methyl isobutyl ketone.

In recent years, hypervalent iodine(iii) compounds, such as [bis(trifluoroacetoxy)iodo]benzene (PIFA) and iodosylbenzene (PhIO), and iodine(v) compounds, such as iodylbenzene (PhIO_2_), have been extensively used in organic synthesis owing to their low toxicity, ready availability and easy handling.^21^ In organic chemistry, they are frequently used for the development of green methodologies for various oxidative transformations.^22–27^ Hypervalent iodine reagents have been prospected for selective oxidation of benzylic halides to corresponding carbonyl compounds in good yields. However, oxidative transformation of CMF to corresponding alcohol (HMF) in good yields is not realized so far under mild one pot reaction condition using hypervalent iodine reagents. Therefore, we envisioned exploring the feasibility of utilizing the oxidation potential of iodosylbenzene (PhIO) in oxidation of biomass derived CMF to afford HMF in good yields.

This manuscript describes an efficacious protocol for production of CMF directly from spent aromatic biomass *via* chemical processing in a biphasic reaction media consisting of concentrated HCl and chloroform in the presence of NaCl using a sealed pressure glass reactor. No such attempt has been made earlier for waste to wealth recovery of value added products from spent aromatic biomass, and the methodology appears to be economically attractive due to high selectivity in product formation with minimal by products, short reaction time, and use of NaCl as a cheap catalyst under mild reaction condition. The manuscript also describes a novel one pot synthesis of HMF from biomass derived CMF by the application of PhIO as a reagent under mild oxidative reaction conditions.

## Experimental

### Materials and method

All the reactions were executed in one-hour oven dried glassware at 100 °C. All reagents and solvents including sodium chloride (NaCl), dichloroethane (DCE), dichloromethane (DCM), chloroform, 5-hydroxymethylfurfural (HMF), and ethyl-α-d-glucoside, ethyl-β-d-glucoside, levulinic acid (LA), ethyl levulinate (EL), formic acid (FA), d-xylose, d-glucose, and hydrochloric acid (HCl) were purchased from Sigma Aldrich (Merck KGaA) as analytical grade and used as received without any modifications. Biomass was powdered by using a universal grinding machine (Kinematica PX-MFC 90 D).

Thin layer chromatography (TLC) was performed on 60 F254 silica gel pre-coated aluminium plates and revealed with either a UV lamp (*λ*_max_ = 254 nm) or a specific colour reagent (Dragendorff reagent or iodine vapours) or by spraying with methanolic-H_2_SO_4_ solution and subsequent charring by heating at 100 °C. Infrared spectra recorded as Nujol mulls in KBr pallets. Monosaccharides, carboxylic acids, HMF, CMF, and ethyl levulinate were detected and quantified by HPLC (Agilent, Model 1260) using Hi-Plex H (Agilent) analytical column (300 mm length, 8 mm porosity). Gas chromatographic analysis was performed on Thermo Scientific GC-MS (Model-Trace 1300 gas chromatograph) with auto sampler and ISQ LT mass spectrometer with columns: HP-5MS (0.25 × 30 m), film thickness 1.0 μm. ^1^H and ^13^C NMR were recorded at 500 and 125 MHz, respectively. Chemical shifts given in ppm downfield from internal TMS; *J* values in Hz.

### Feedstock preparation and determination of fractional composition

Spent aromatic biomass was collected after the on-farm hydro-distillation of aromatic crops at Center of Innovative and Applied Bioprocessing, Mohali (CIAB), Punjab state, India. Shade dried biomass (moisture content of <7% wt) was subjected to reduction of particle size to 0.5 mm (30 mesh) using a grinding machine. Primary composition of spent aromatic biomass (such as carbohydrate sugars, lignin, and ash) was determined following the Standard National Renewable Energy Laboratory (NREL) protocol.

### Pre-treatment of spent aromatic waste

Powdered biomass was pre-treated with *p*-cymene sulphonic acid (*p*-CSA), a Brønsted acid synthesised from d-limonene as a renewable feed stock from solid citrus waste, under autoclave condition following the protocol reported in literature.^28^ The pre-treated biomass was separated from hydrolysate simply by filtration followed by drying in a hot air oven at 45–50 °C.

### Synthesis of 5-(chloromethyl)furfural (CMF) from pre-treated biomass

Pre-treated spent aromatic biomass (1.0 g), concentrate HCl (5 mL), chloroform (15 mL) and NaCl (50 mg) were loaded on a thick-walled high-pressure glass reactor (Ace Glass, USA) of 120 mL capacity sealed (back) with silicone rubber. The reaction mixture was heated at 100 °C in an oil bath for 1 h. After time elapsed, the sealed glass tube was removed from oil bath and subsequently cooled to room temperature by the application of cold water. The reaction mixture was centrifuged at 8000 rpm for 20 min. Pallet was collected and stored for further use. Organic phase was separated from reaction liquid while the aqueous phase was repeatedly washed with chloroform. After pooling, the organic phase was subjected to GC-MS analysis for detection and quantification of CMF. Aqueous phase was separately analysed by HPLC for detection and quantification of carbohydrates (*e.g.*, glucose, fructose, arabinose *etc.*) and their degradation products (HMF, levulinic acid, formic acid, acetic acid *etc.*).

Concentration of organic phase under reduced pressure resulted in a dense yellow liquid which was further purified by column chromatography to afford a light yellow oil in good yield (∼26% with respect to pre-treated biomass) and purity (∼98%). ^1^H NMR (500 MHz, CDCl_3_): *δ* 9.64 (s, 1H), 7.22 (d, *J* = 3.6, 1H), 6.60 (d, *J* = 3.6, 1H), 4.62 (s, 2H). ^13^C NMR (125 MHz, CDCl_3_): *δ* 177.76, 156.07, 152.86, 121.80, 111.96, 36.52.

### One pot synthesis of HMF from CMF

A RB flask containing CMF (100 mg) along with DMSO and water in ratio of 4 : 1 (v/v) was taken. The reaction mixture was added with freshly prepared PhIO^29^ (455 mg, 3.0 equiv.) and heated at 55–60 °C for 3 h. After time elapsed, the reaction was allowed to cool at room temperature followed by removal of solvent under reduced pressure. Reaction mixture was extracted with ethyl acetate followed by washing with water and brine. Organic layer was separated and dried over anhydrous sodium sulphate. Concentration of the organic phase under reduced pressure furnished HMF as a light yellow liquid (91 mg, ∼90% yield).

### Detection and quantification of HMF and CMF by GC-MS analysis

The compounds CMF and HMF were validated using gas chromatography (GC) method stated as: a column HP-5MS (0.25 × 30 m), film thickness, 1.0 μm; helium at flow rate of 1.0 mL min^−1^ was used as a carrier gas. Oven temperature gradient was performed from 60 °C to 210 °C at 3 °C min^−1^ ramp rate and 1 min hold time at 210 °C; then 210 °C to 280 °C at 20 °C min^−1^ ramp rate with 5 min hold time at 280 °C. Samples were injected with 1 μL with split ratio of 1 : 20 and inlet temperature at 250 °C. Mass spectra were scanned from 50 to 400 amu mass range with electron impact ionization energy at 70 eV, source temperature of 280 °C and mass transfer line temperature of 280 °C.

### Quantitative HPLC analysis of spent aromatic biomass hydrolysate

Xylose, glucose, arabinose, acetic acid, HMF, formic acid and levulinic acid in the hydrolysate of spent aromatic biomass were quantified by high-performance liquid chromatography (Thermo Scientific UltiMate 3000 HPLC) using the analytical standards of d-xylose, d-glucose, d-arabinose, acetic acid, HMF, formic acid and levulinic acid under chromatographic conditions stated as: Agilent Hi-Plex H column (300 mm length; 8 μm porosity), RI detector operated at 60 °C, mobile phase of 5 mM H_2_SO_4_, flow rate of 0.7 mL per min, refractive index (RI) as detector at 60 °C and run time of 40 min. Quantitative estimations of xylose, glucose, arabinose, acetic acid, HMF, formic acid, ethyl-α-d-glucoside, ethyl-β-d-glucoside, and levulinic acid in hydrolysate was made using a calibration curve drawn by plotting concentration of known standards *versus* peak area values from the RI chromatograms.

### Recovery of lignin from hydro-char

The lignin from the hydro-char was obtained by boiling it in 2% aq. NaOH solution for 2 h at 100 °C. Centrifugation of the reaction liquid at 8000 rpm for 20 min followed by separation and neutralization of the black liquid with 0.5 N H_2_SO_4_ solution resulted in the precipitation of the lignin at pH 3.0. The precipitate was filtered out, washed with water and dried in a hot air oven to afford lignin in crystalline state. Yield of lignin was calculated using the following formulae.
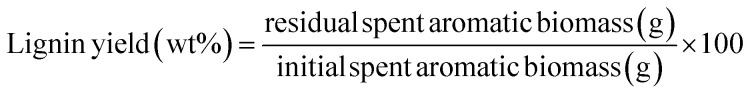


## Results and discussion

This study has been carried out to explore a divergent approach for the production of HMF in high yield and purity from spent aromatic waste (such as lemongrass, citronella grass, and palmarosa fibres) derived CMF as a renewable feed stock. The approach recruited includes: (a) pre-treatment of spent aromatic waste; (b) production of CMF from pre-treated biomass; (c) recovery of lignin from hydro-char; (d) synthesis of HMF from CMF using iodosyl benzene (PhIO).

### Pre-treatment of spent aromatic waste

The complex recalcitrant structure of the spent aromatic waste prevents it from being efficiently hydrolysed, hence, a pre-treatment stage is usually essential to overcome the biomass recalcitrance for having effective biomass utilization. Several biomass pre-treatment approaches so far have been explored, such as physical (milling, energy irradiation), physicochemical (liquid hot water, steam explosion), chemical (acid, alkali, organosolv), and biological pretreatments (bacteria mediation).^30^ In literature reports, the bio-based *p*-cymenesulfonic acid (*p*-CSA) containing a sulfonic (–SO_3_H) group has been cited as an efficient alternative to petrochemical derived toluene sulfonic acid (*p*-TSA) for the hydrolysis of cellulosic material in aqueous medium. *p*-CSA can be produced directly from d-limonene as a renewable feed stock form citrus waste.^28^ A selective and near complete (>90%) hydrolysis of xylan polysaccharide in spent aromatic biomass by *p*-CSA under autoclave condition has been prospected.^31,32^ The method shows efficacy for xylose production with minimum side products. Accordingly, the spent aromatic waste was treated with aqueous solution of *p*-CSA in an autoclave for 90 min. After time elapsed, reaction mixture was cooled followed by filtration to separate the hydrolysate from unreacted biomass. HPLC analysis of the hydrolysate demonstrated a high concentration of xylose (162 mg, >90% yield with respect to hemicellulose; ∼16% yield with respect to initial spent aromatic biomass) while other side products *e.g.* glucose, arabinose, CH_3_COOH were observed only in traces. The hydrolysate was *in vaccuo* concentrated, wherefrom, xylose was recovered by precipitation with alcohol. Collection, washing and crystallization of precipitate afforded xylose in good purity.

### Production of CMF from pre-treated biomass

Mascal group reported the first pathway for CMF production from carbohydrates (*e.g.*, glucose, sucrose, or cellulose, *etc.*) in good yields.^8^ However, application of this method in the processing of biomass resulted in the formation of furfural as a co-product due to an analogous conversion of the hemicellulosic C5 sugars. Therefore, additional steps were required for isolation of products from reaction mixture which may increase the cost of downstream processing. The pre-treatment of biomass before thermochemical processing for CMF production appears promising in minimizing the formation of co-products in reaction liquid. The pre-treated biomass obtained after hydrolysis of spent aromatic waste with *p*-CSA exhibits improved characteristics such as low ash and high content of cellulose and lignin. Therefore, in a typical run, 1.0 g pre-treated biomass and concentrate HCl in a ratio of 1 : 1 (w/v), NaCl (2.5 wt%), and CHCl_3_ (total solid–liquid ration 1 : 20 w/v) were loaded to a thick-walled high pressure glass reactor. After 1 h refluxing at 100 °C in an oil bath, the reactor was cooled to room temperature. The reaction mixture was subjected to centrifugation, the pallet was collected and dried separately in a hot air oven. The organic phase was separated form reaction liquid and dried over anhydrous Na_2_SO_4_. GC-MS analysis of organic phase established the formation of CMF as a major product (60 mg, ∼6% yield with respect to pre-treated biomass) while glucose, ethyl-α,β-glucosides, levulinic acid, 5-HMF, and ethyl levulinate were observed only in traces.

In order to access the yield further, we optimize the reaction condition with respect to temperature, reaction time, effect of solvents, amount of conc. HCl, and loadings of NaCl. Without NaCl, the liquid products of pre-treated biomass under thermal processing in presence of HCl alone were many, with low yield and selectivity. GC-MS analysis of the organic phase displayed the presence of CMF and ethyl levulinate in low yields, meanwhile the HPLC analysis of aqueous phase (see ESI Fig. S1†) displayed the formation of monosaccharides and cellulose degradation products *e.g.* glucose, levulinic acid, 5-HMF, formic acid, ethyl-α,β-glucosides *etc.* A rise in HCl loading did not cause a substantial change in the selectivity and yield of products. Upon addition of NaCl, a significant enhancement in the concentration of CMF was observed in the organic phase after 1 h reaction time (Table 2). This indicated that the NaCl promotes both the yield of, and selectivity to CMF under heating condition. In HPLC analysis of aqueous phase, a decreasing glucose concentration at increasing NaCl loadings was attributed to its efficient conversion to CMF which being insoluble to aqueous phase readily diffuses to organic phase and detected therefrom by GC analysis (see ESI Fig. S2†). Thus, the yield of CMF increased with increasing the amount of NaCl at fixed HCl loading in the reactions. Highest CMF concentration in organic phase was detected at 0.05 equivalent of NaCl (5 wt% with respect to pre-treated biomass). A further increase in the amount of NaCl did not cause an enhancement of CMF yield due to near complete degradation of cellulose occurring in the pre-treated biomass.

**Table 2:** Detection and quantification of compounds in aqueous and organic phase

Entry^a^^,^^b^	NaCl (wt%)	Cellulose degradation products^c^ (wt%)									
		Organic phase				Aqueous phase					
		Furfural	EL	HMF	CMF	Glucose	EADG	EBDG	LA	FA	HMF
1	BLK	0.01	0.35	0.19	14.52	6.77	0.44	0.81	0.23	0.10	0.34
2	2.5	0.06	0.68	0.22	18.18	5.21	0.17	0.12	0.57	0.18	0.16
3	5.0	0.06	1.40	0.14	25.87	2.15	0.23	0.05	1.34	0.64	0.22
4	10	0.07	1.41	0.13	25.37	2.68	0.21	0.06	1.00	0.48	0.23
5	20	0.09	0.66	0.13	24.23	3.07	0.31	0.05	1.81	0.76	0.19
6	40	0.04	0.92	0.06	21.08	2.17	0.19	0.08	1.43	0.47	0.20

a Reaction condition: biomass, 1.0 g; concentrate (35%) HCl, 5 mL; chloroform, 15 mL; temperature, 100 °C; reaction time, 1 h.

b Solid–liquid ratio, 1 : 20 in all sets of reaction.

c BLK, blank; EL, ethyl levulinate; EADG, ethyl-α-d-glucopyranoside; EBDG, ethyl-β-d-glucopyranoside; LA, levulinic acid; FA, formic acid.

Mascal group^10–12^ has earlier established the crucial role of chlorinated solvents in the synthesis of CMF from carbohydrates under thermal condition. Therefore, the effect of organic solvents on the yield of CMF from biomass was investigated by carrying out reactions in presence of CHCl_3_, DCM, and DCE as the solvent system. The results suggested that the ability of organic solvents to extract out CMF from aqueous phase is highly desired. CMF was detected in low yields when DCM was used as reaction solvent. Similarly, DCE displayed moderate efficacy in CMF extraction from aqueous phase. The solvent CHCl_3_ performed well due the high ability of CMF extraction from aqueous phase, hence, established as the solvent of choice for the reaction.

**Fig. 1:**
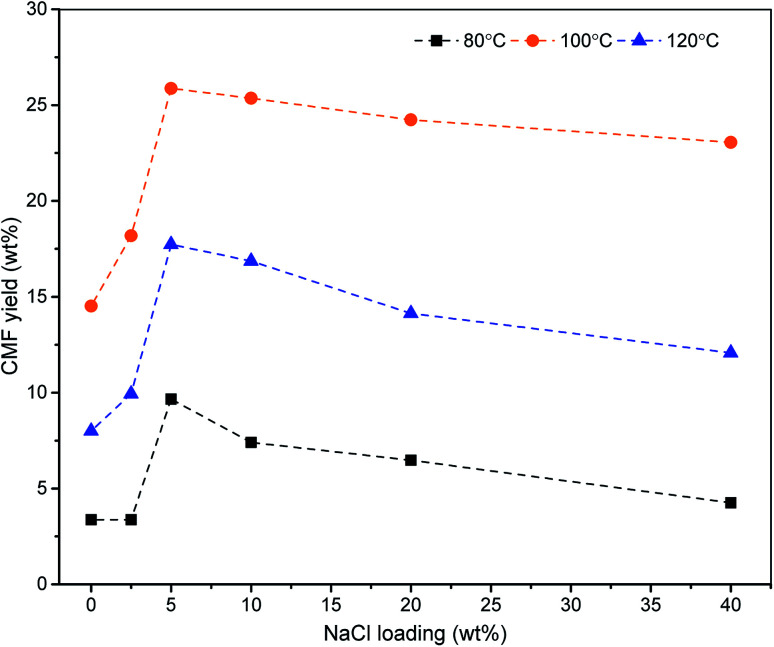
Effect of temperature on CMF yields *via* solvo-thermal processing of biomass.

**Fig. 2:**
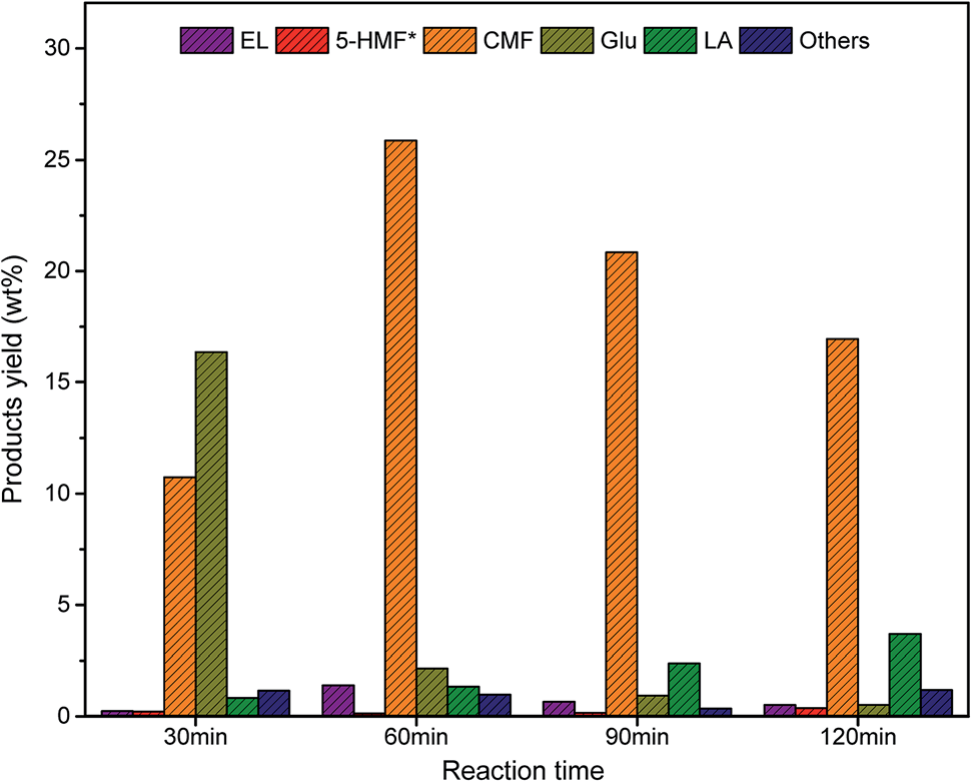
Effect of reaction time on CMF yields *via* solvo-thermal processing of biomass.

The effect of temperature on the yield of CMF was also studied at constant loading of NaCl and HCl. Lowering of the temperature below 100 °C caused a rapid decrease in the CMF yield. Maximum yield of CMF from the biomass could be obtained at 100 °C. An increase in reaction temperature beyond 100 °C lowered the CMF yield due to the formation of unidentified soluble products (Fig. 1). Similarly, the reaction time was also found to have a profound impact on cellulose degradation and transformation of resulting monosaccharide to CMF. Lowering of the reaction time from 1 h at constant temperature (100 °C) resulted in a lot of glucose remained unreacted in reaction liquid. Processing of biomass under prolonged reaction time beyond 1 h at 100 °C resulted into an increased concentration of LA and polymeric materials in the hydrolysate (Fig. 2). Since, the designed methodology shows high selectivity towards CMF (Table 3), the reaction predominantly produces CMF which being insoluble in aqueous phase, simply diffuses to organic phase and recovered therefrom in good yields *via* concentration under the reduced pressure. Pure CMF (∼98%) was produced by column chromatography followed by validation through GC analysis (see ESI Fig. S3†) and characterization by spectroscopic techniques such as NMR (see ESI Fig. S4 and S5†) and MS (see ESI Fig. S6†).

**Table 3:** Selectivity of products in biomass hydrolysate by HPLC analysis

SN	Biomass	Products selectivity							Glucose (wt%)	Conversion^c^ (wt%)
		Furfural	EL	HMF^a^	CMF	LA	FA	Others^b^		
1	Palmarosa	0.18	4.36	1.10	80.56	4.20	1.98	7.60	2.15	71.08
2	Lemon grass	0.22	4.84	1.62	80.27	5.23	2.62	5.21	1.30	69.43
3	Citronella grass	0.19	3.88	1.78	79.55	6.11	3.21	5.28	1.16	66.38

a Both in aqueous and organic phases.

b Others such as ethyl-α,β-glucopyranosides and unreacted glucose.

c Based on glucan content in spent biomass.

The cellulose degradation products, *e.g.*, glucose, LA, FA, and ethyl glucosides *etc.* were all together detected in a very small quantity (∼2–3%) in aqueous phase. Further, dissolving the residue (solid left after the reaction) in methanol established no black tar formation. Hence, neither black tar formation nor gasification could be observed in processing of biomass with NaCl and HCl under the optimized reaction conditions. As evident from Table 2, the highest concentration of CMF from pre-treated aromatic biomass (1.0 g) was obtained with a condition of 100 °C temperature, 1 h reaction time, 0.05 equivalent NaCl, 1 : 5 w/v loading of conc. HCl and 1 : 15 w/v loading of CHCl_3_.

**Fig. 3:**
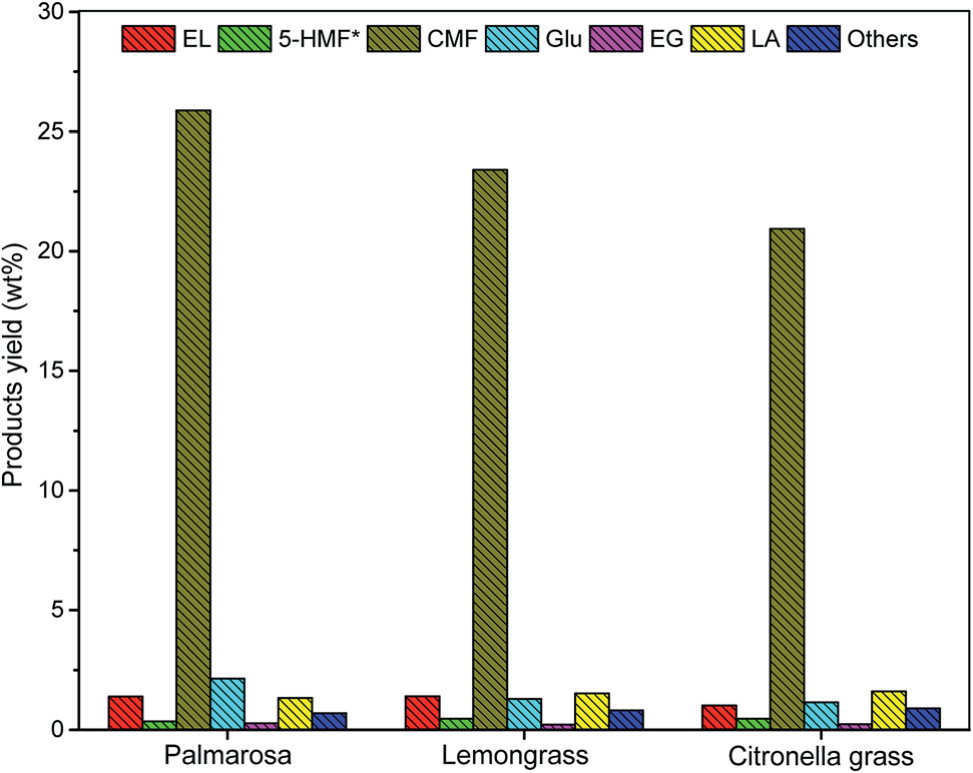
Comparative yield of cellulose degradation products from spent aromatic waste under the optimized reaction condition.

Once the production of CMF from pre-treated palmarosa biomass was established using NaCl and HCl under solvo-thermal condition, the methodology was successfully extended over the other pre-treated biomass such as lemongrass and citronella grass, wherein, the results demonstrated a near complete conversion of cellulose to CMF (Fig. 3). A plausible mechanism involves the acid hydrolysis of polymeric cellulose into glucose by the activity of mineral acid in presence of NaCl. A subsequent isomerization of glucose generates fructose which on dehydration results into the formation of 5-hydroxymethylfurfural (HMF) as intermediate that undergoes chlorination to form CMF in good yields.^10^

### Recovery of lignin from hydro-char

The hydro-char recovered as pallet after the synthesis of CMF from palmarosa biomass was boiled in a dilute NaOH solution. The aqueous phase (black liquid) was removed and neutralized separately by dilute H_2_SO_4_ solution, wherein, the lignin present in the black liquid was precipitated at pH 3.0. Separation, washing, and drying of precipitate furnished lignin (∼14% yield) which was further characterization by FT-IR, XRD and EDS analysis.

The FT-IR spectrum of the isolated lignin exhibited absorption bands indicative to the presence of phenolic and alcoholic groups (3500 cm^−1^), –CH_2_–H and <svg xmlns="http://www.w3.org/2000/svg" version="1.0" width="10.400000pt" height="16.000000pt" viewBox="0 0 10.400000 16.000000" preserveAspectRatio="xMidYMid meet"><metadata> Created by potrace 1.16, written by Peter Selinger 2001-2019 </metadata><g transform="translate(1.000000,15.000000) scale(0.011667,-0.011667)" fill="currentColor" stroke="none"><path d="M80 1160 l0 -40 40 0 40 0 0 -40 0 -40 40 0 40 0 0 -40 0 -40 40 0 40 0 0 -40 0 -40 40 0 40 0 0 -40 0 -40 40 0 40 0 0 -40 0 -40 40 0 40 0 0 -40 0 -40 40 0 40 0 0 80 0 80 -40 0 -40 0 0 40 0 40 -40 0 -40 0 0 40 0 40 -40 0 -40 0 0 40 0 40 -40 0 -40 0 0 40 0 40 -40 0 -40 0 0 40 0 40 -80 0 -80 0 0 -40z M560 520 l0 -40 -40 0 -40 0 0 -40 0 -40 -40 0 -40 0 0 -40 0 -40 -40 0 -40 0 0 -40 0 -40 -40 0 -40 0 0 -40 0 -40 -40 0 -40 0 0 -40 0 -40 -40 0 -40 0 0 -40 0 -40 80 0 80 0 0 40 0 40 40 0 40 0 0 40 0 40 40 0 40 0 0 40 0 40 40 0 40 0 0 40 0 40 40 0 40 0 0 40 0 40 40 0 40 0 0 80 0 80 -40 0 -40 0 0 -40z"/></g></svg> C–H stretching (2800–2900 cm^−1^), carbonyl function (1600–1780 cm^−1^) and aromatic ring (∼1400 cm^−1^). Absorption bands corresponding to syringyl and guaiacyl residues were observed between 1300–1100 cm^−1^ (see ESI Fig. S7†). X-ray diffraction pattern of lignin showed many sharp diffraction peaks in which the peaks centred at 19.19° and 32.31° were typical to pure lignin (see ESI Fig. S8†). Other sharp peaks indicated a certain level of crystallinity due to the impurities and/or small crystalline fragments. Elemental composition of the isolated lignin was determined by energy-dispersive X-ray spectroscopy (EDS) analysis which established the elemental composition: C, 71.33%; O, 21.94% as presented in (see ESI Fig. S9†). Other elements such as Na (3.19%), Cl (1.79%), and S (1.03%) were also detected as impurities due to the use of dilute NaOH and H_2_SO_4_ in the process of lignin isolation.

The mass balance and the carbon balance of product distribution from spent aromatic biomass was calculated based on the experiments performed in this paper, results are summarized in Table 4. The main products were xylose, CMF, and lignin. In the designed process, most of lignin present in the spent biomass was degraded (∼10–15%). About ∼10–20% weight loss was attributed to the presence of solvent extractives and residual volatiles. It is noticeable that the mass balance closures were not close to 100% because some degradation products from the hemicelluloses and cellulose were not detected in HPLC analysis. Additionally, some solids were adsorbed on to the filter paper and wall of funnel during the filtration process, therefore, could not be collected. Moreover, the designed methodology produces CMF in high selectivity which being highly soluble in chloroform, is isolated easily from the reaction mixture. We carried out a weight based analysis to check the formation of gases during the processing of spent aromatic biomass for production of CMF under the optimized reaction condition. Results of screening test carried out at different temperatures such as 80, 100, and 120 °C as summarized in Table 5, established that no significant loss in the weight of reaction mixture before and after the reaction was observed, hence, ruled out the formation of gases *e.g.* CO_2_, CO, CH_4_*etc.*

**Table 4:** Mass balance of products produced from pre-treated biomass

SN	Type of biomass	Qty. of biomass^a^ (g)	Liquefied products (wt%)		Residue^b^ (wt%)	Lignin (wt%)	Biomass conversion (wt%)	Carbon balance^c^ (wt%)
			Organic phase	Aqueous phase				
1	Palmarosa	1.0	27.47	4.42	29.0	14.21	58.35	91.08
2	Lemon grass	1.0	25.12	4.04	27.5	13.47	59.64	83.14
3	Citronella	1.0	22.29	4.03	27.0	13.23	57.61	79.31

a Biomass loading at conc. HCl (5 mL), chloroform (15 mL), 100 °C temperature, reaction time 1 h.

b Unreacted biomass recovered in reaction.

c Carbon balance after degradation of cellulose.

**Table 5:** Weight based analysis for the formation of gases during the biomass processing

Entry^a^	Reaction temperature (°C)	Reaction mixture weight^b^ (g)		Weight lose (wt%)	Gaseous products (CO, CO_2_, CH_4_)
		Before reaction	After reaction		
1	80	269.0	268.87	0.05	ND
2	100	270.7	270.65	0.02	ND
3	120	266.9	266.53	0.14	ND

a Reaction condition: biomass, 1.0 g; concentrate (35%) HCl, 5 mL; chloroform, 15 mL; reaction time, 1 h.

b Weight of reaction mixture including the sealed glass reactor.

### Synthesis of HMF from CMF using iodosyl benzene (PhIO)

**Fig. 4:**
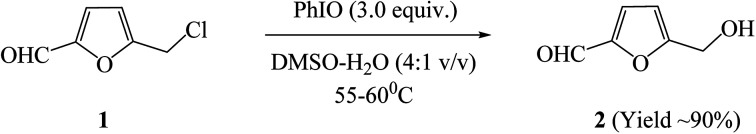
Synthesis of HMF from CMF using PhIO.

In the past several decades, hypervalent iodine chemistry has witnessed prosperous development in the field of organic synthesis by the use of the hypervalent iodine reagents as mild oxidants.^13^ Therefore, we investigated the feasibility of utilizing the oxidising properties of iodosylbenzene (PhIO) for an efficient conversion of CMF to HMF in good yields and selectivity. Accordingly, the CMF was reacted with PhIO in presence of DMSO and water as solvent system under the mild heating condition. HMF in the reaction mixture was validated by GC-MS analysis which demonstrated a complete conversion of CMF to HMF (Fig. 4).

The reaction was investigated with respect to effect of temperature, effect of reaction time, and the ratio of DMSO and water as solvent system. The reaction carried out in DMSO as a solvent alone under the anhydrous condition did not cause the formation of HMF even at the prolonged reaction time (24 h) that established the essentiality of water for a successful reaction. The reaction performed in presence of H_2_O as solvent alone could result in the low yield of HMF probably due to the substrate insolubility in the water. Hence, the use of two-phase solvent system comprising of water and organic solvent was anticipated, wherein, the mixtures of H_2_O and water immiscible solvents, *i.e.*, dichloromethane, chloroform, ethyl acetate, *etc.*, did not work due to the high affinity of CMF to remain in the organic phase. Similarly, the reaction could not be successful with water miscible solvents, *e.g.*, methanol, ethanol, acetonitrile, *etc.* Considering the DMSO as a high-polarity water miscible solvent, we performed the oxidation of CMF with PhIO in a mixture of DMSO–water under mild heating condition, wherein, the peak corresponding to HMF was significantly detected in GC-MS analysis. Increasing the reaction temperature beyond 60 °C resulted into the formation of levulinic acid as a side product. Moreover, the concentration of water decisively influenced the efficacy of oxidation as no reaction was observed with excess of water when used along with DMSO as solvent. Thus, different gradients of DMSO/H_2_O were screened for an efficient oxidation of CMF to HMF by using PhIO, where a mixture of DMSO/water in a ratio of 4 : 1 (v/v) demonstrated the best results. Also, high loadings of PhIO was required for completion of reaction. Thus, under the optimized reaction condition, the CMF (1.0 equiv.) was reacted with PhIO (3.0 equiv.) in presence of DMSO and water in a ratio of 4 : 1 (v/v) as solvent system under mild heating for 3 h to afford HMF as a light yellow liquid in good yields (∼90%) and purity (see ESI Fig. S10 and S11†).

**Fig. 5:**
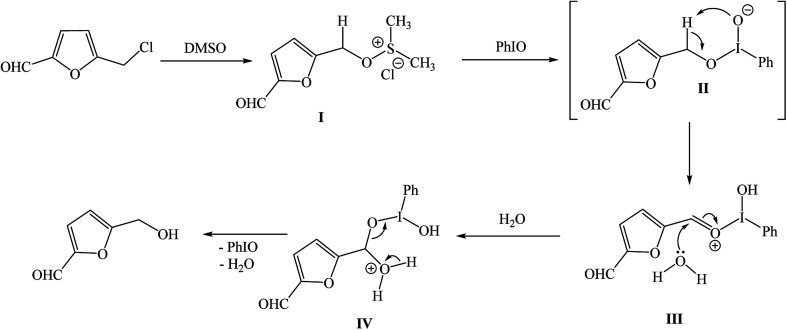
Reaction mechanism for conversion of CMF to HMF under the oxidative reaction condition.

Although, a detailed understanding of the mechanism for such an oxidative transformation of CMF to HMF will require additional studies, however, it is assumed that the initial reaction of CMF with DMSO may lead to the formation of an addition product I which reacts with PhIO and generates intermediate II that decomposes to a more stable intermediate III. Action of water molecule cause an easy release of product HMF *via* the decomposition of intermediate IV (Fig. 5).

## Conclusion

This manuscript describes a method for chemical processing of an abundant and underutilized biomass which does not have high value application and generally disposed of or burned openly, thereby, causes environmental pollution. Spent aromatic biomass may significantly be used as a renewable feed stock for production of CMF which being hydrophobic in nature, may be recovered from aqueous medium in high yields and purity. CMF can be potentially used as a versatile chemical for production of furfurals, LA, EL, EADG *etc.* while xylose being a precursor for many valuable chemicals and an abundant building unit of hemicellulose, can be specifically produced in this approach to add an economic advantage.

To date no scalable method is available for production of HMF from raw biomass. High sensitivity of HMF to the acidic conditions results in the formation of numerous side products which impose difficulties in its production from biomass. Therefore, a new method for synthesis of HMF from the biomass derived CMF under oxidative condition using hypervalent iodine reagent PhIO has also been investigated in this manuscript. Use of PhIO as an oxidant provides easy access to HMF from CMF in high yields and selectivity under the mild reaction condition. Thus, this approach would be promising and commercially more acceptable method for the production of CMF, HMF and their derivatives from spent aromatic residues.

## Conflicts of interest

The authors declare no conflict of interest.

## Supplementary Material

RA-010-D0RA09310F-s001
