# Supplementary material for: A divergent approach for the synthesis of (hydroxymethyl)furfural (HMF) from spent aromatic biomass-derived (chloromethyl)furfural (CMF) as a renewable feedstock
Source: RSC Adv. 2020 Dec 21;10(73):45081–9. doi: 10.1039/d0ra09310f (PMC9058557; doi:10.1039/d0ra09310f)
Supplement: RA-010-D0RA09310F-s001 [file RA-010-D0RA09310F-s001.pdf]

Electronic Supplementary Material (ESI) for RSC Advances.
This journal is © The Royal Society of Chemistry 2020


**A divergent approach for synthesis of (hydroxymethyl)furfural (HMF) from spent aromatic biomass derived (chloromethyl)furfural (CMF) as a renewable feed stock**

Mangat Singh,[a,b] Nishant Pandey,[a,b] Bhuwan B. Mishra[a]*

[a]Center of Innovative and Applied Bioprocessing (CIAB), Sector 81 (Knowledge City), S.A.S. Nagar, Mohali-140306, Punjab, India

[b]Department of Chemistry, Faculty of Science, Panjab University, Chandigarh-160014, India

---

## Table of Contents


| | | |
|---|---|---|
| **1.** | **Figure S1.** HPLC chromatogram of hydrolysate obtained *via* processing of pre-treated biomass with conc. HCl at 100 °C | 2 |
| **2.** | **Figure S2.** HPLC chromatogram of hydrolysate obtained *via* processing of pre-treated biomass with conc. HCl and NaCl at 100 °C | 3 |
| **3.** | **Figure S3.** GC chromatogram of CMF | 4 |
| **4.** | **Figure S4.** Mass spectrum of CMF | 5 |
| **5.** | **Figure S5.** $^{1}H$ NMR spectrum of CMF | 6 |
| **6.** | **Figure S6.** $^{13}C$ NMR spectrum of CMF | 7 |
| **7.** | **Figure S7.** FT-IR of standard (S-LIN) and isolated lignin (SB-LIN) | 8 |
| **8.** | **Figure S8.** XRD analysis of isolated lignin | 9 |
| **9.** | **Figure S9.** Elemental composition of isolated lignin by EDS analysis lignin | 10 |
| **10.** | **Figure S10.** GC chromatogram of HMF | 11 |
| **11.** | **Figure S11.** Mass spectrum of HMF | 12 |


Number of pages: 12

Number of figures: 11

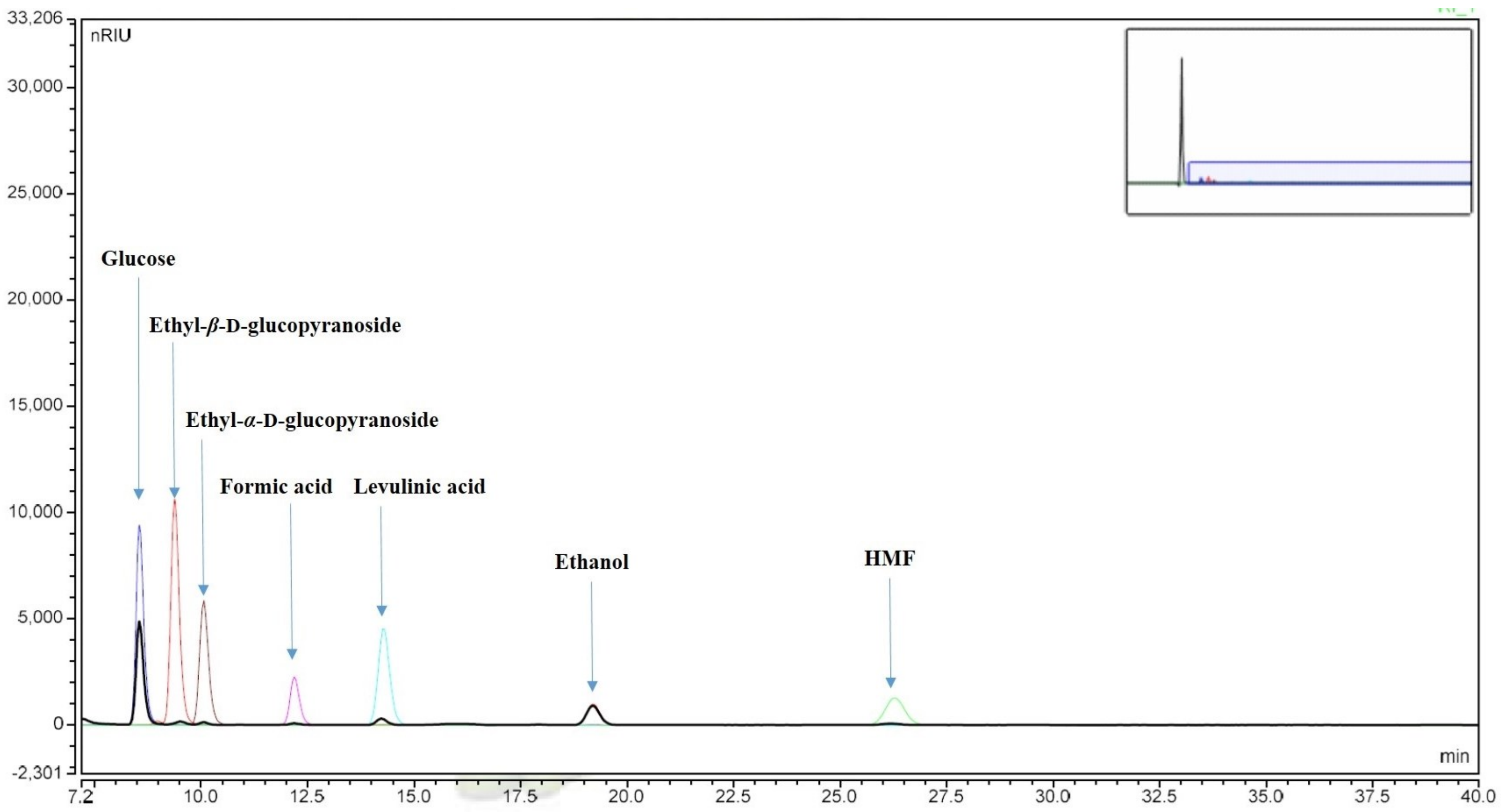


**Figure S1.** HPLC chromatogram of hydrolysate obtained *via* processing of pre-treated biomass with conc. HCl at 100 °C

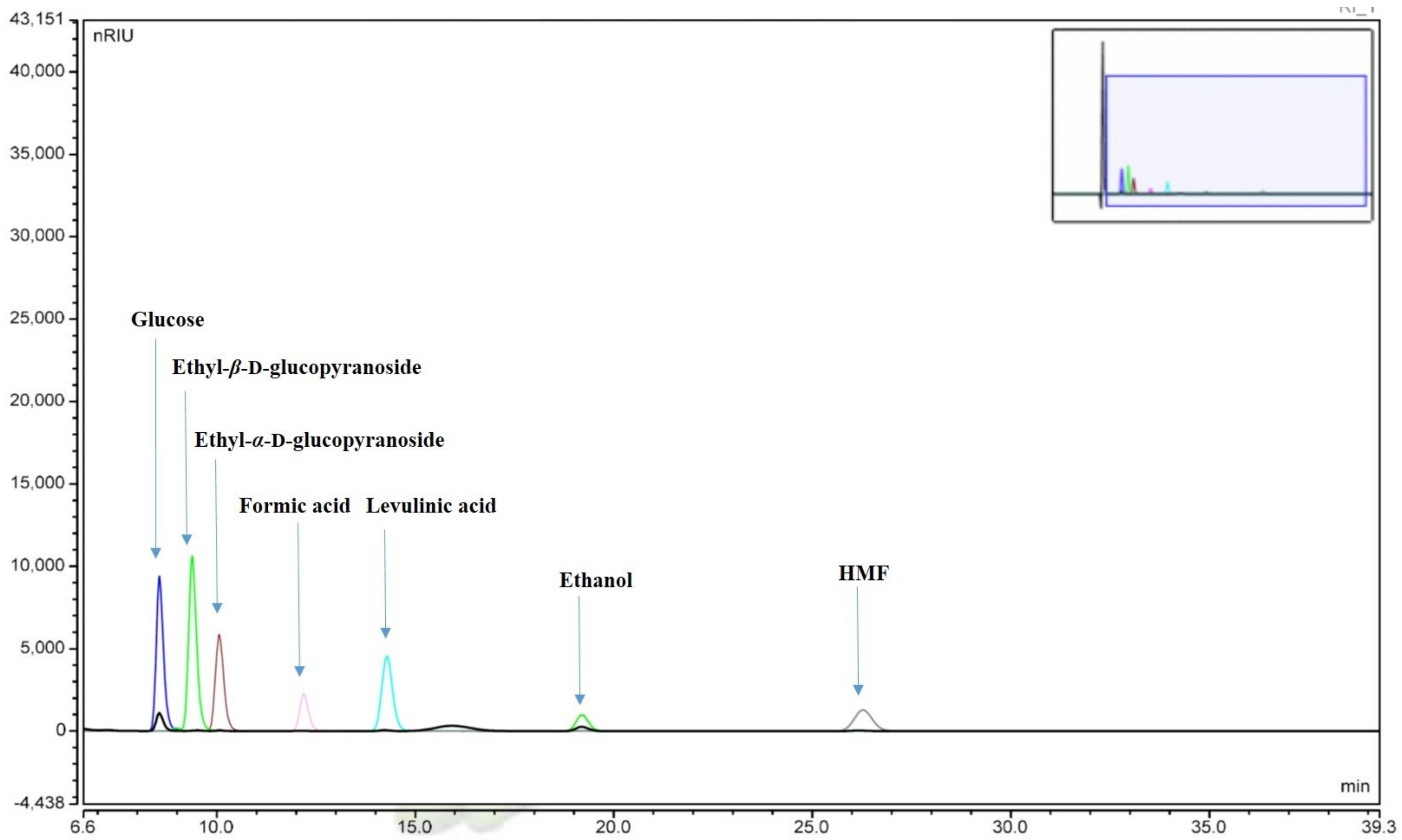


**Figure S2.** HPLC chromatogram of hydrolysate obtained *via* processing of pre-treated biomass with conc. HCl and NaCl at 100 °C

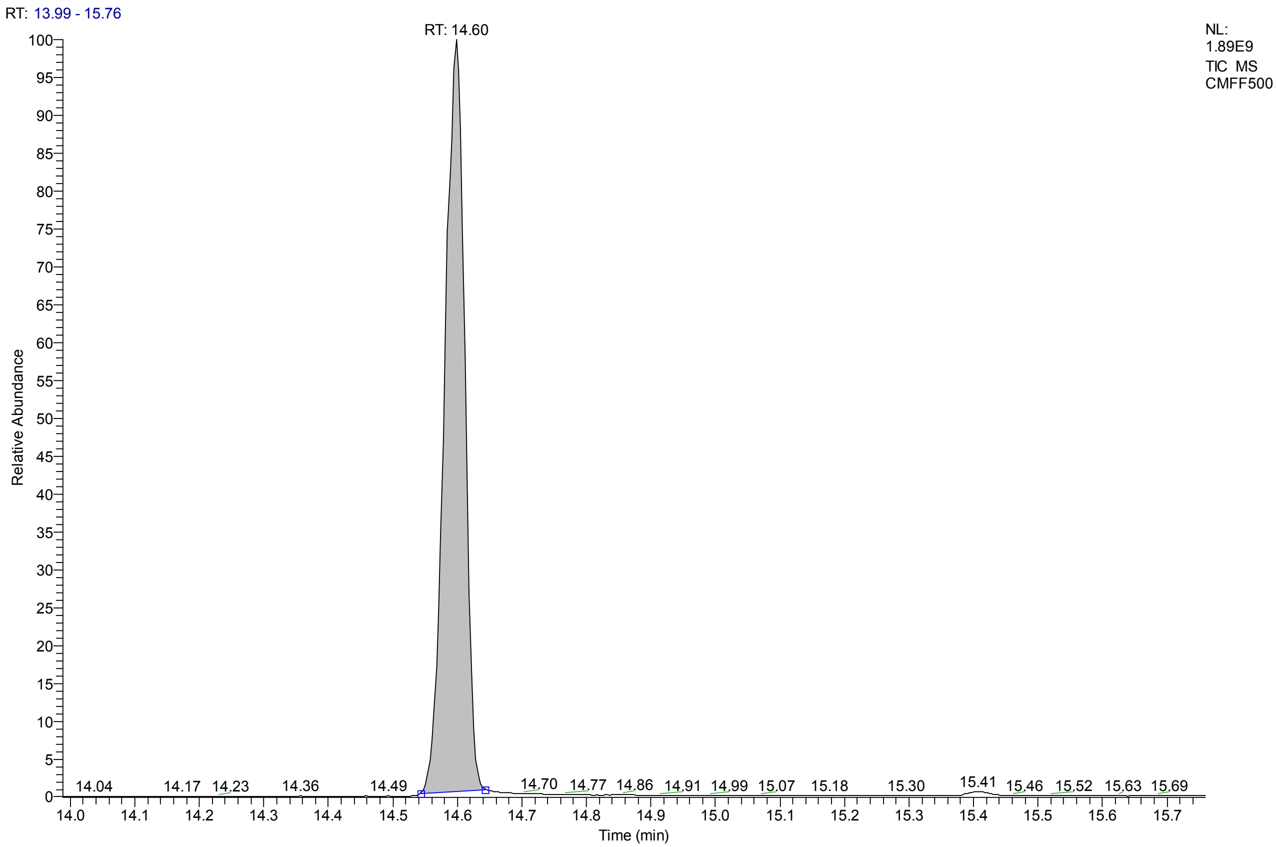


**Figure S3.** GC chromatogram of CMF

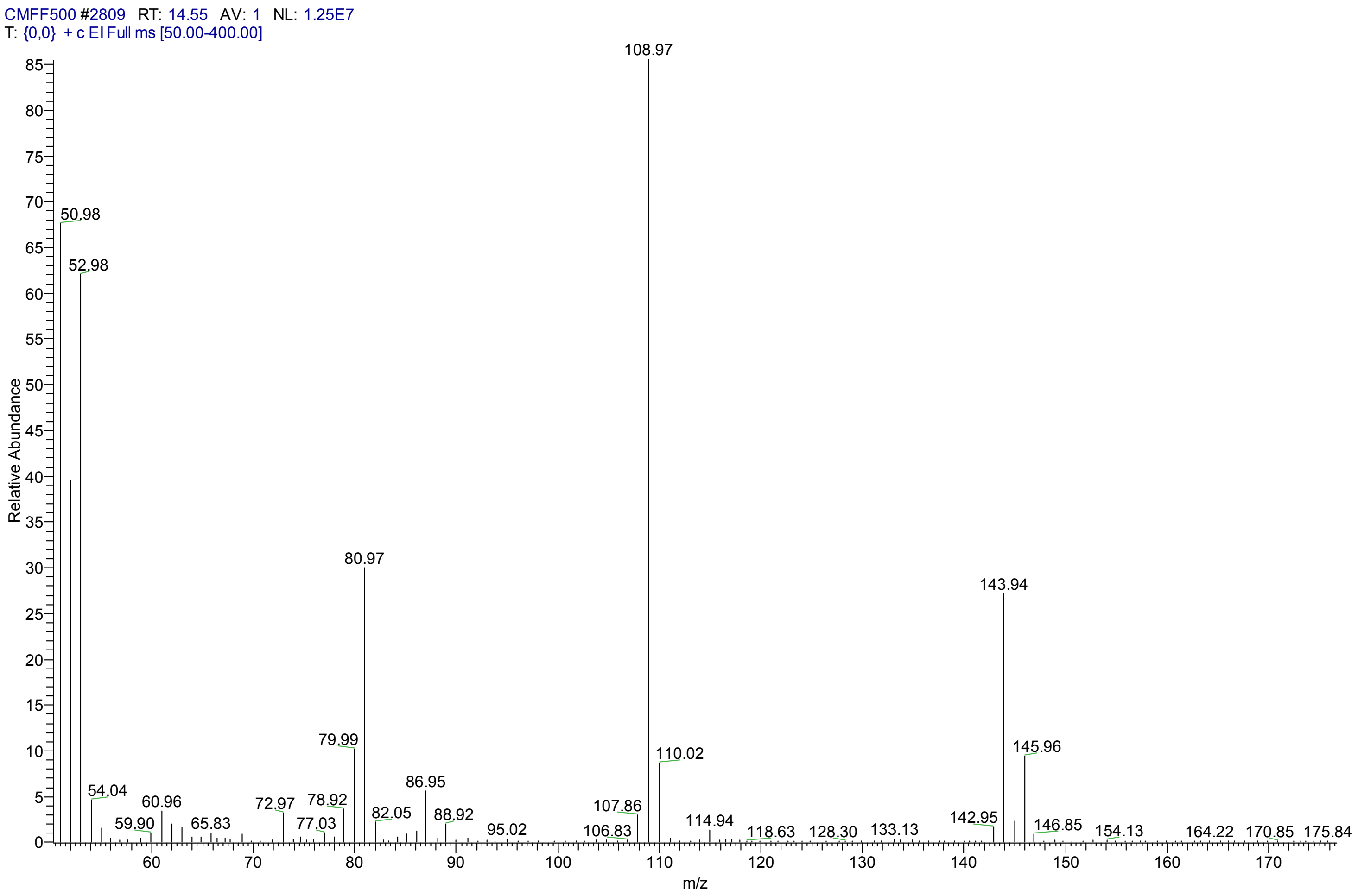


**Figure S4.** Mass spectrum of CMF

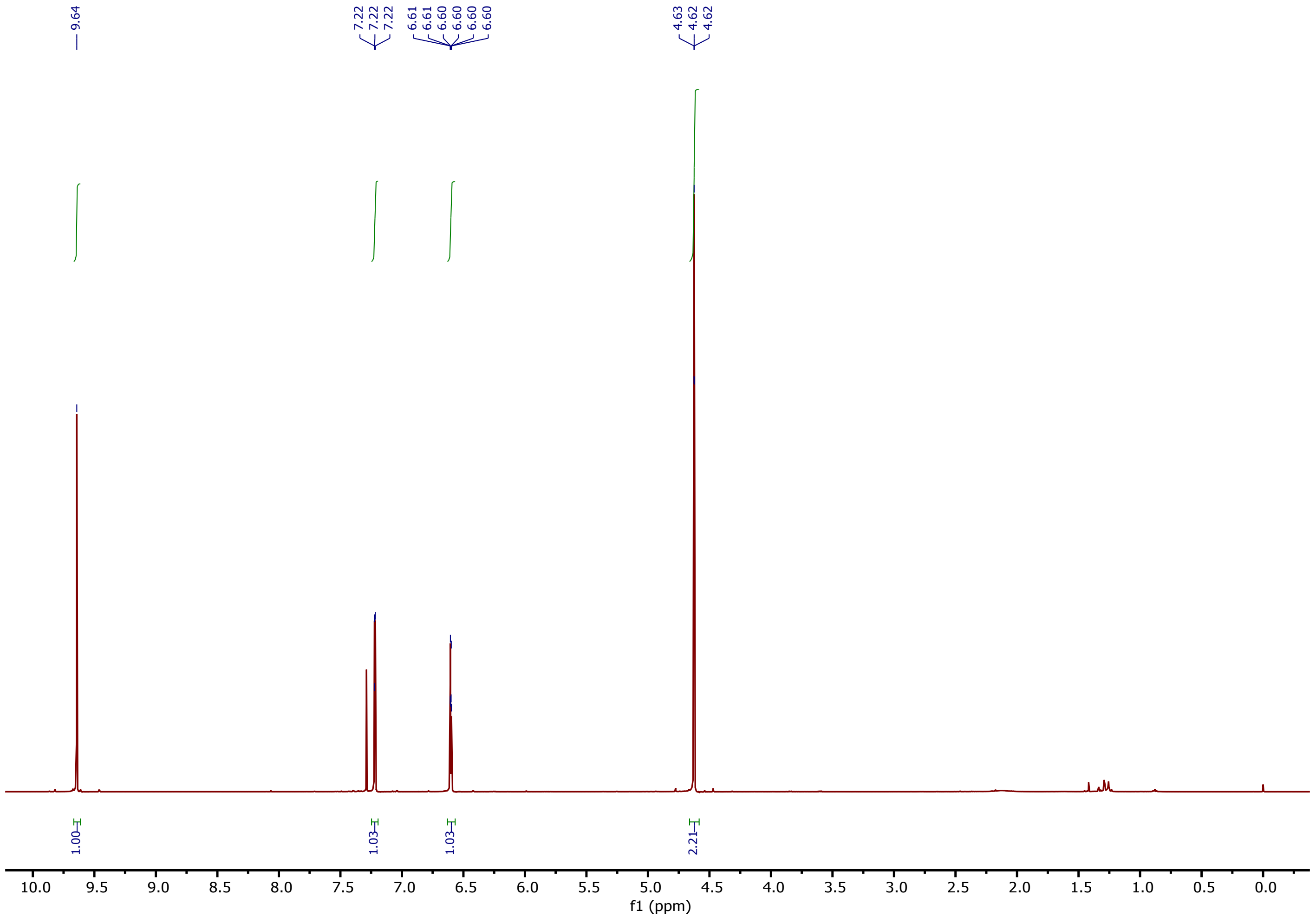


**Figure S5.** $^{1}$H NMR spectrum of CMF

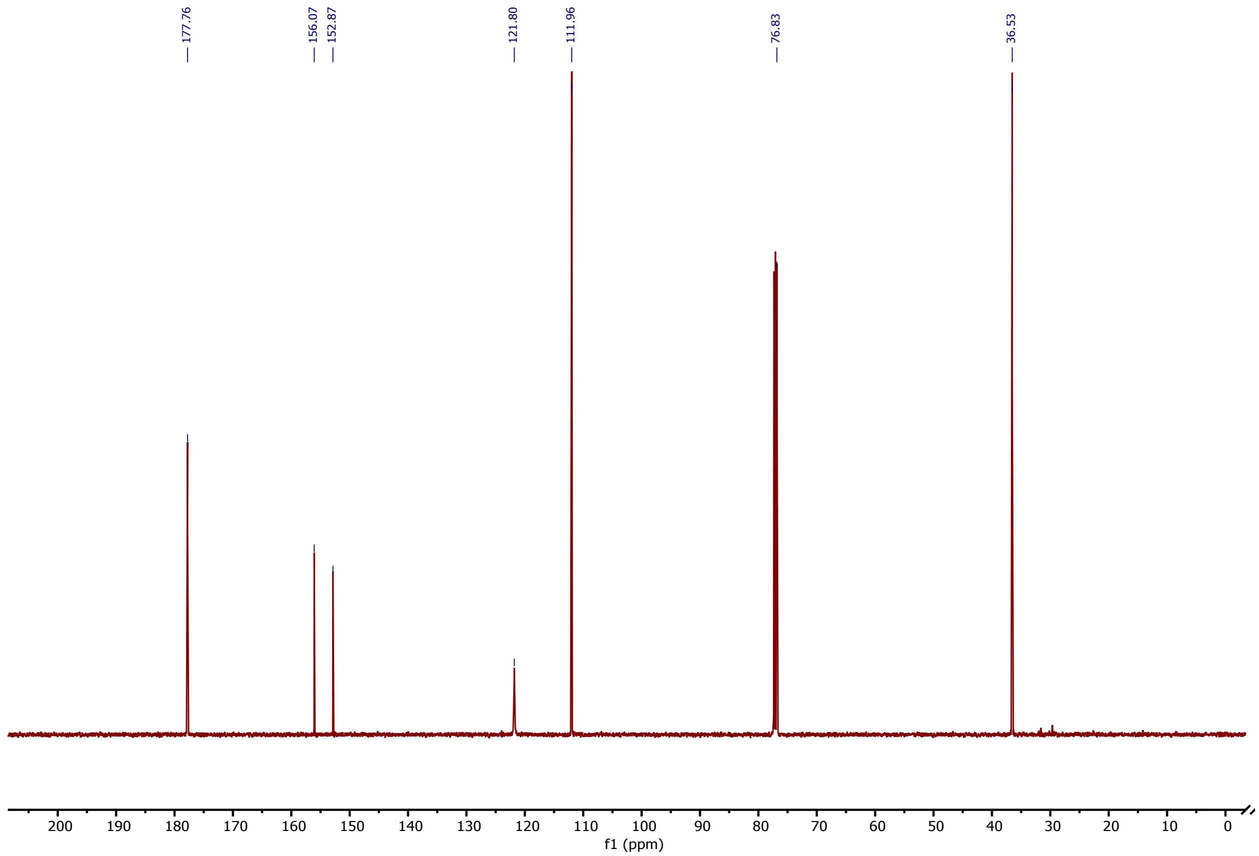


**Figure S6.** $^{13}$C NMR spectrum of CMF

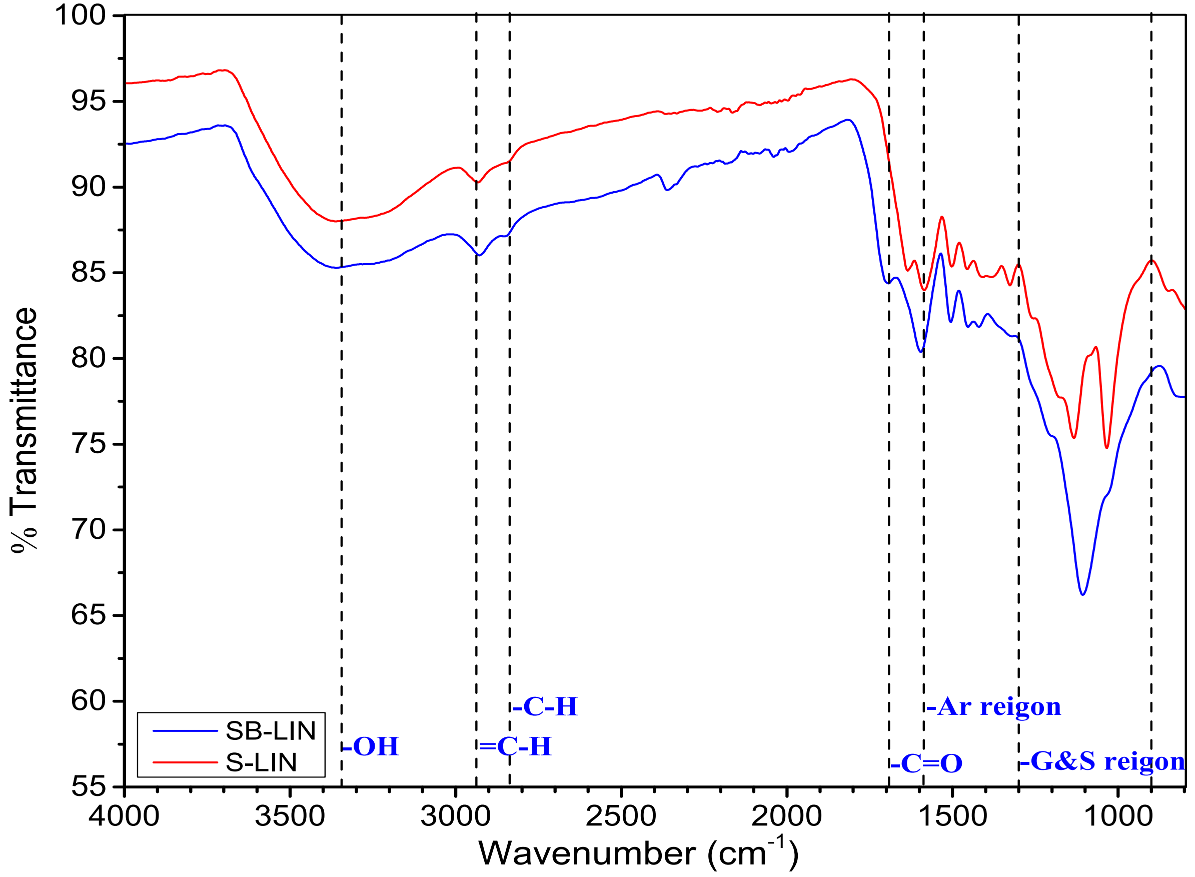


**Figure S7.** FT-IR of standard (S-LIN) and isolated lignin (SB-LIN)

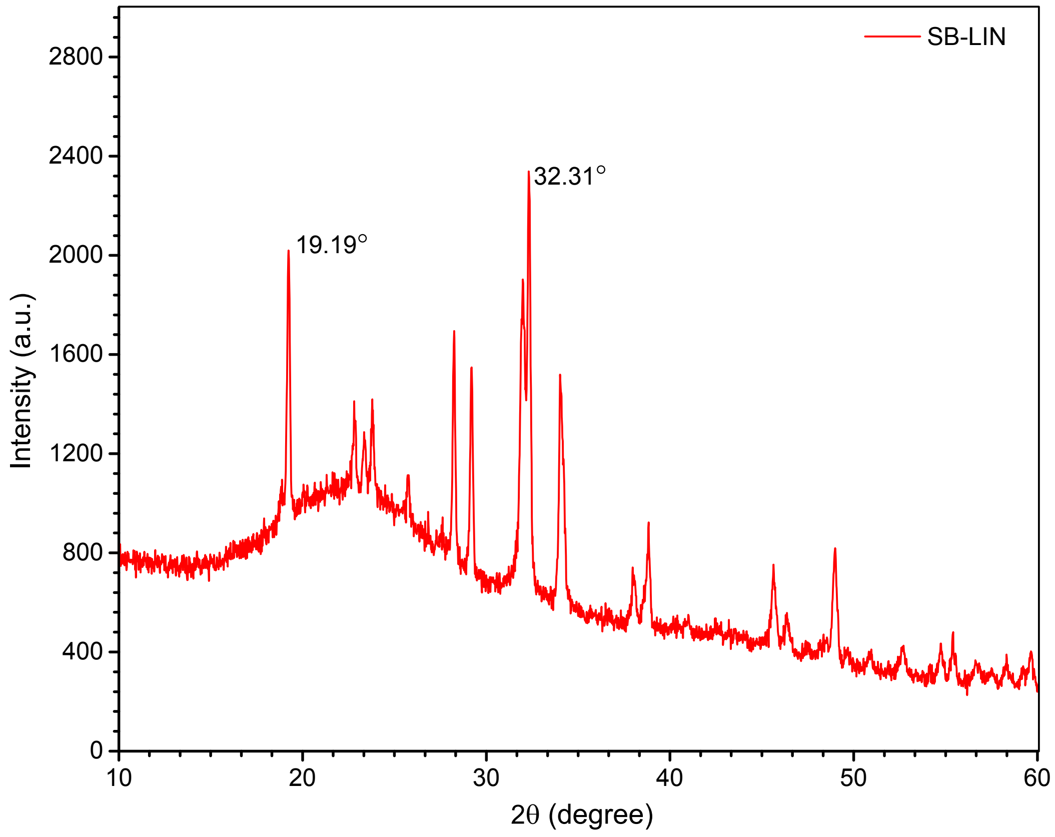


**Figure S8.** XRD analysis of isolated lignin

## Application Note

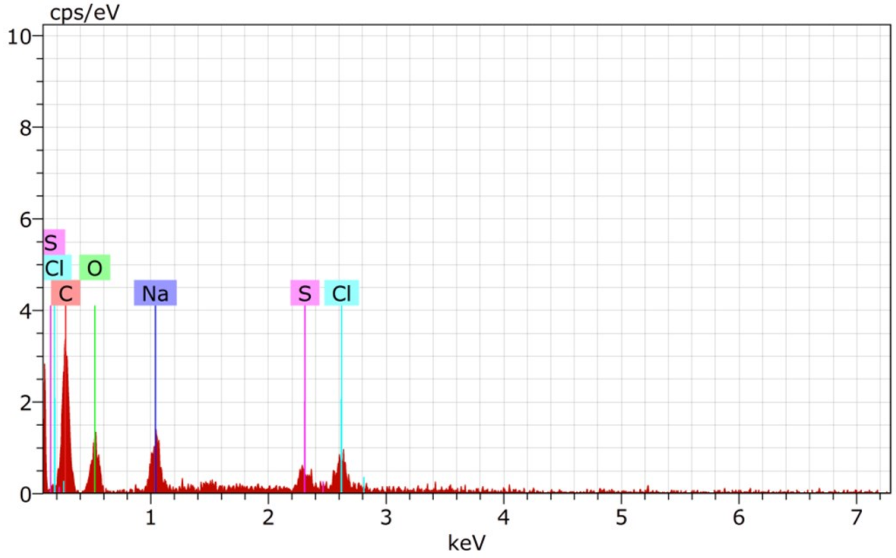


Spectrum: test 6397

| Element | Series | unn. C [wt.%] | norm. C [wt.%] | Atom. C [at.%] | Error (3 Sigma) [wt.%] |
|---|---|---|---|---|---|
| Carbon | K-series | 36.08 | 61.46 | 71.33 | 24.24 |
| Oxygen | K-series | 14.78 | 25.18 | 21.94 | 13.62 |
| Sodium | K-series | 3.78 | 6.45 | 3.91 | 1.14 |
| Chlorine | K-series | 2.67 | 4.55 | 1.79 | 0.63 |
| Sulfur | K-series | 1.39 | 2.37 | 1.03 | 0.43 |
| | Total: | 58.70 | 100.00 | 100.00 | |

**Figure S9.** Elemental composition of isolated lignin by EDS analysis lignin

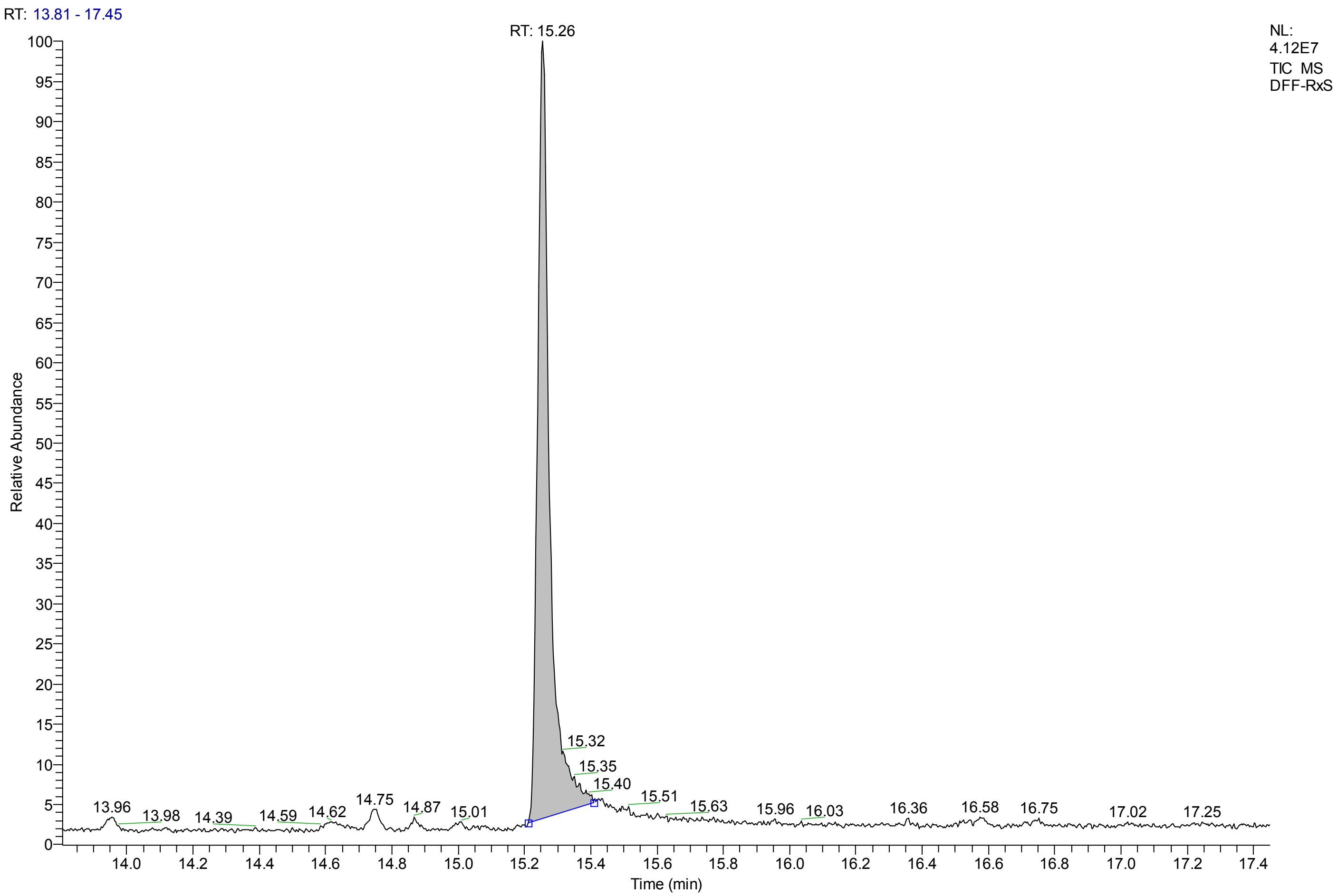


**Figure S10.** GC chromatogram of HMF

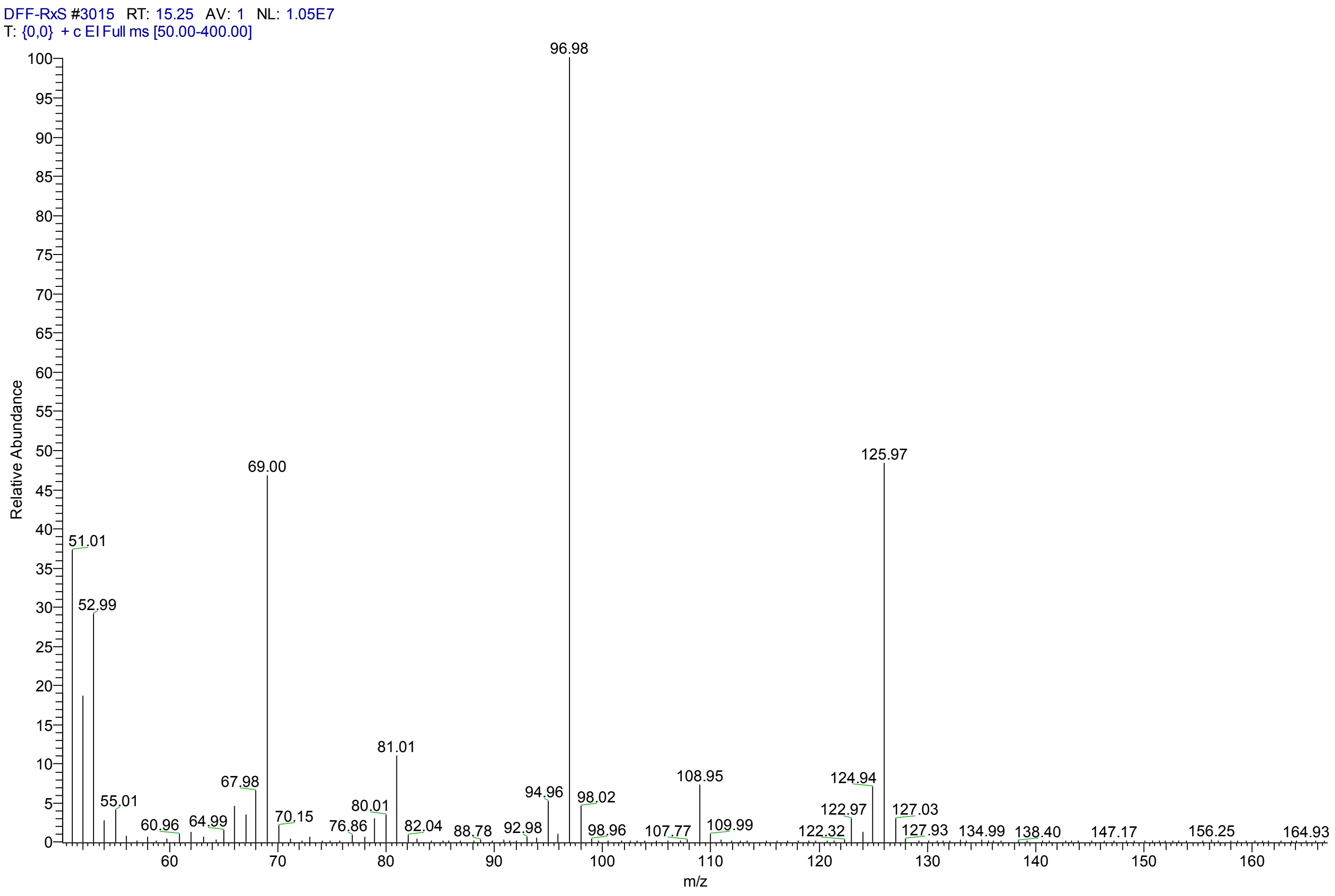


**Figure S11.** Mass spectrum of HMF
